# Supplementary material for: Terminal uridyltransferase 7 regulates TLR4-triggered inflammation by controlling Regnase-1 mRNA uridylation and degradation
Source: Nat Commun. 2021 Jun 29;12:3878. doi: 10.1038/s41467-021-24177-7 (PMC8241994; doi:10.1038/s41467-021-24177-7)

Supplementary Figure 1, Related to Fig. 1

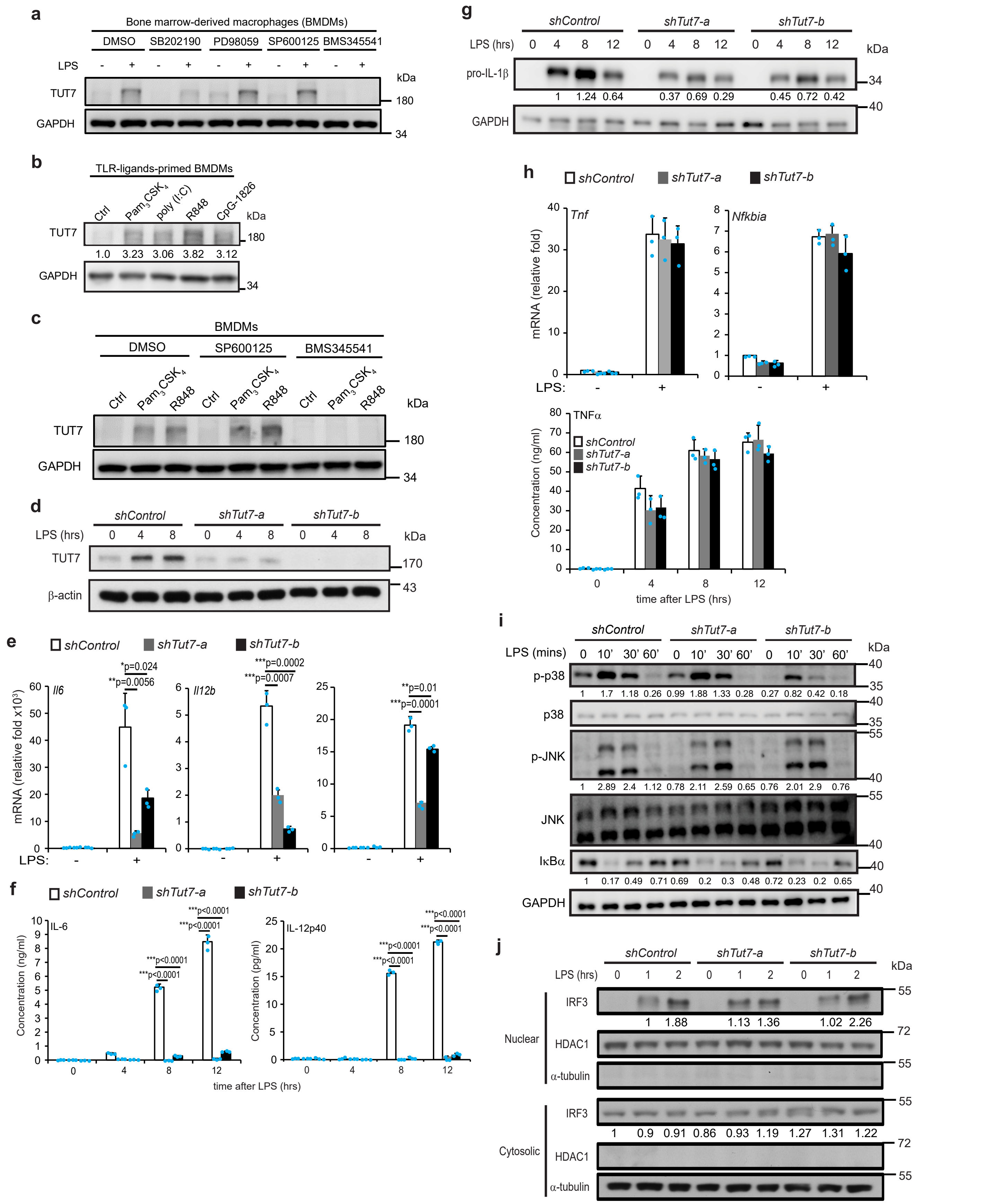

Supplementary Figure 1. Depletion of TUT7 affects the production of LPS-induced inflammatory cytokines in RAW 264.7 macrophages. (a) Wild-type BMDMs were pre-treated with the indicated kinase inhibitors, including SB202190 (p38 inhibitor), PD98059 (ERK inhibitor), SP600125 (JNK inhibitor), and BMS345541 (IKK inhibitor) for 30 min and then incubated with 100 ng/ml LPS for 8 hr. The expression of TUT7 was analyzed by immunoblotting. (b) Wild-type BMDMs were treated with different TLR ligands, including Pam_3CSK_4 (TLR1/2, 1 μg/ml), poly(I:C) (TLR3, 30 μg/ml), R848 (TLR7/8, 1 μg/ml) and CpG-1826 (TLR9, 1 μM), for 8 hr. The expression of TUT7 was analyzed by immunoblotting. (c) Wild-type BMDMs were pre-treated with JNK (SP600125) and IKK (BMS345541) inhibitors followed by stimulation of Pam_3CSK_4 (TLR1/2, 1 μg/ml) and R848 (TLR7/8, 1 μg/ml) for 8 hr. The expression of TUT7 was analyzed by immunoblotting. (d-j) RAW 264.7 macrophages were infected with lentiviruses encoding control shRNA (*shControl*) or *shTut7* before stimulation with 100 ng/ml of LPS. Total RNAs were obtained from cells 4 hr after LPS stimulation. The expression of the indicated mRNAs and TUT7 protein was measured by RT-qPCR and immunoblotting, respectively (d, e, h). Cultural media were collected at the indicated time points after LPS treatment, and the amounts of cytokines were analyzed by ELISA (f, h). The expressions of pro-IL-1β, IκBα and phosphorylated-JNK and -p38 in cell lysates were analyzed by immunoblotting (g, i). Control and *shTut7*-exprssing RAW 264.7 macrophages were treated with 100 ng/ml of LPS for the indicated periods of time. The expression of IRF3 in the cytosolic and nuclear fractions were analyzed by immunoblotting. HDAC1 and α-tubulin were used as markers for the nuclear and cytosolic fractions, respectively (j). Data presented are representative of three independent experiments with triplicates in each experiment (error bars, mean ± S.D.). The p values were obtained from two-tailed Student's t-test and are shown in the figure if $p < 0.05$. Source data are provided as a Source Data file.

Supplementary Figure 2, Related to Fig. 1

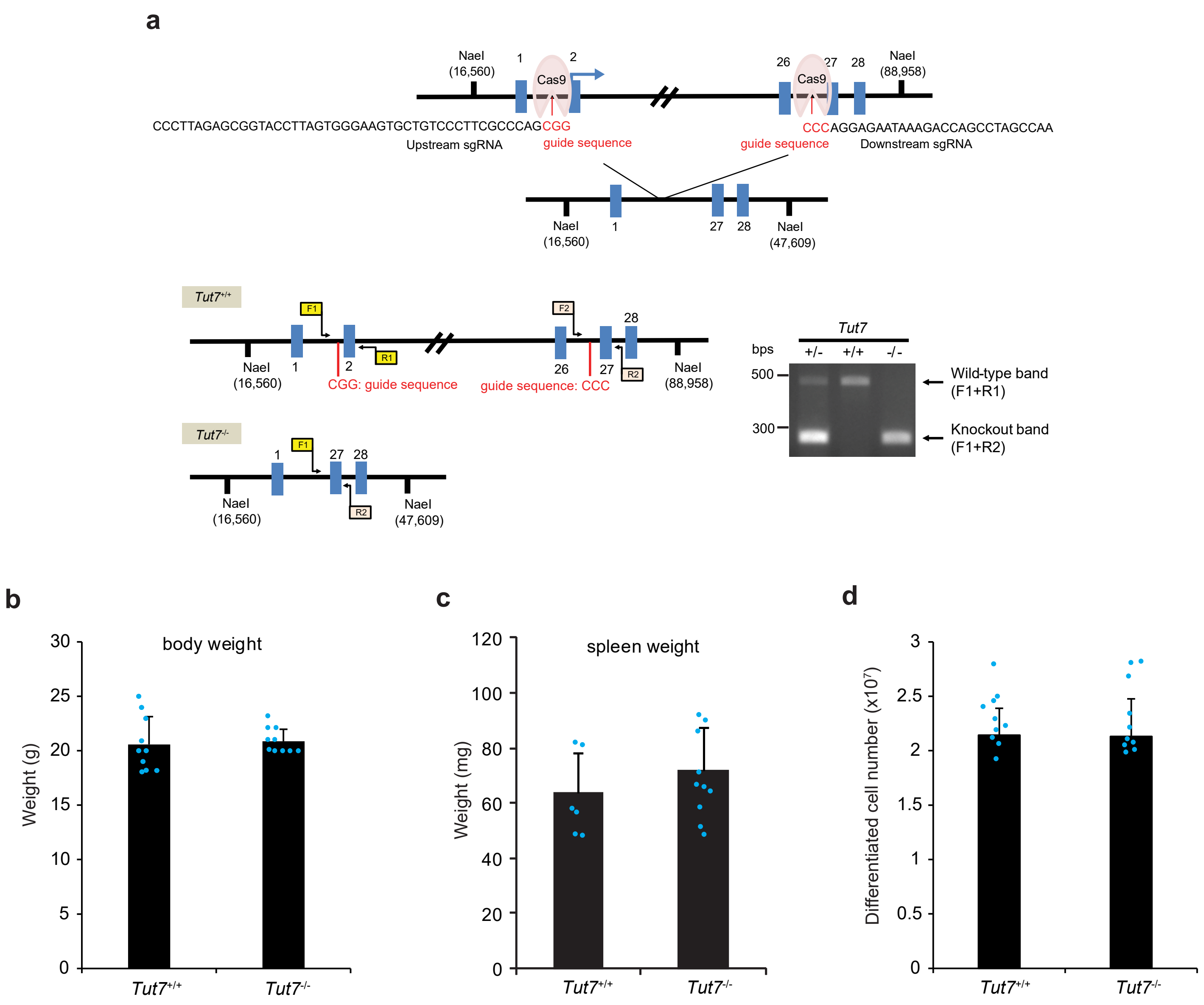

Supplementary Figure 2. Generation of *Tut7*-deleted mice.
(a) Schematic diagram of the mouse wild-type *Tut7* allele, the two target sites on introns 1 and 26 for guide RNAs (gRNAs), and the deleted *Tut7* allele after excision by the CRISPR/Cas9 system. F1 and R1, and F2 and R2 indicate the positions of the two different primer pairs used for PCR genotyping. Genotyping of *Tut7*$^{+/+}$, *Tut7*$^{+/-}$, and *Tut7*$^{-/-}$ mice using their genomic DNA from tails are shown on the bottom right. (b, c) Seven-week old *Tut7*$^{+/+}$ and *Tut7*$^{-/-}$ mice had similar body weight (b), and spleen weight (c). (d) Depletion of TUT7 has no significant effect on *in vitro* differentiation of bone marrow cells into macrophages. Data are mean ± SD for six to ten *Tut7*$^{+/+}$ and *Tut7*$^{-/-}$ mice. Source data are provided as a Source Data file.

Supplementary Figure 3, Related to Fig. 2

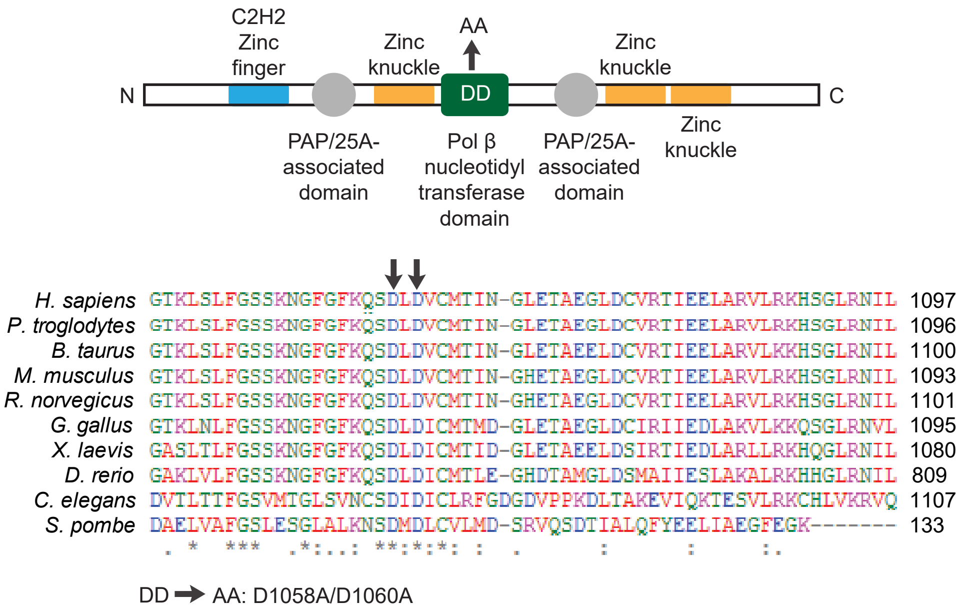

Supplementary Figure 3. The domain organization of TUT7.
Schematic diagram of the functional domains of human TUT7 and sequence alignment of TUT7 among various species. Amino acid sequence alignments were performed using the Clustal Omega program at EMBL-EBI (https://www.ebi.ac.uk/Tools/msa/clustalo). An asterisk (*) denotes an identical residue, a colon (:) denotes conserved substitutions, and a period (.) denotes semi-conserved substitutions. The arrows indicate the two aspartate residues which are critical for its catalytic activity. Source data are provided as a Source Data file.

Supplementary Figure 4, Related to Fig. 2

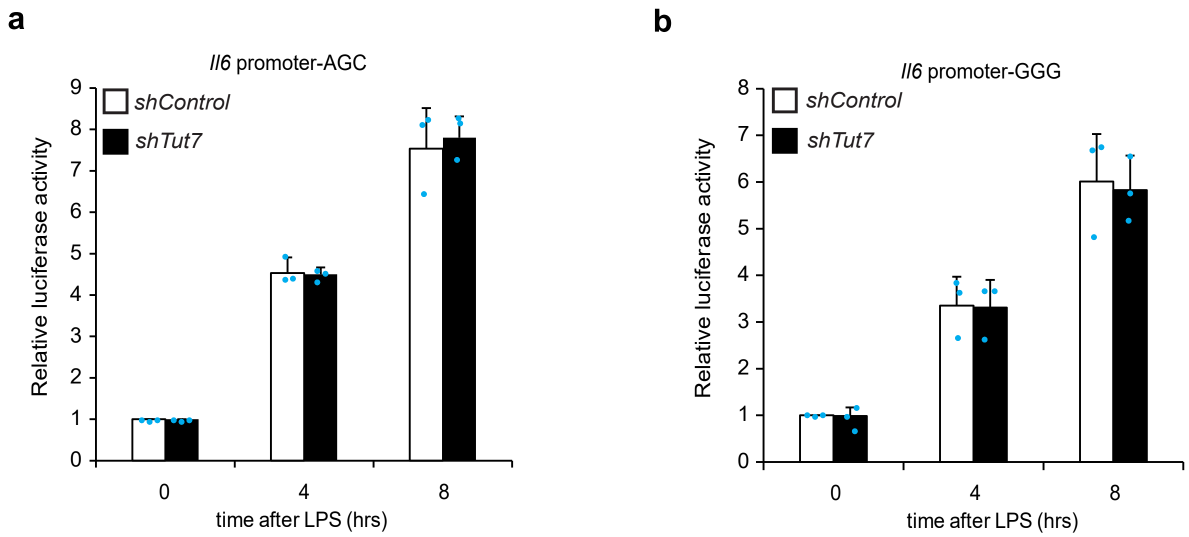

Supplementary Figure 4. TUT7 does not affect IL-6 promoter activity after LPS stimulation.
(a, b) Control and *shTut7*-expressing RAW 264.7 macrophages were co-transfected with *firefly* luciferase reporter plasmids containing *Il6*-AGC (a) or *Il6*-GGG (b) promoter and *TK-renilla* control reporter plasmid. At 48 hr after transfection, cells were treated with LPS (100 ng/ml) for different periods of time. Cells were harvested and the luciferase activity was determined. Data presented are representative of three independent experiments performed in triplicate (error bars, mean ± S.D.). Source data are provided as a Source Data file.

Supplementary Figure 5, Related to Fig. 2

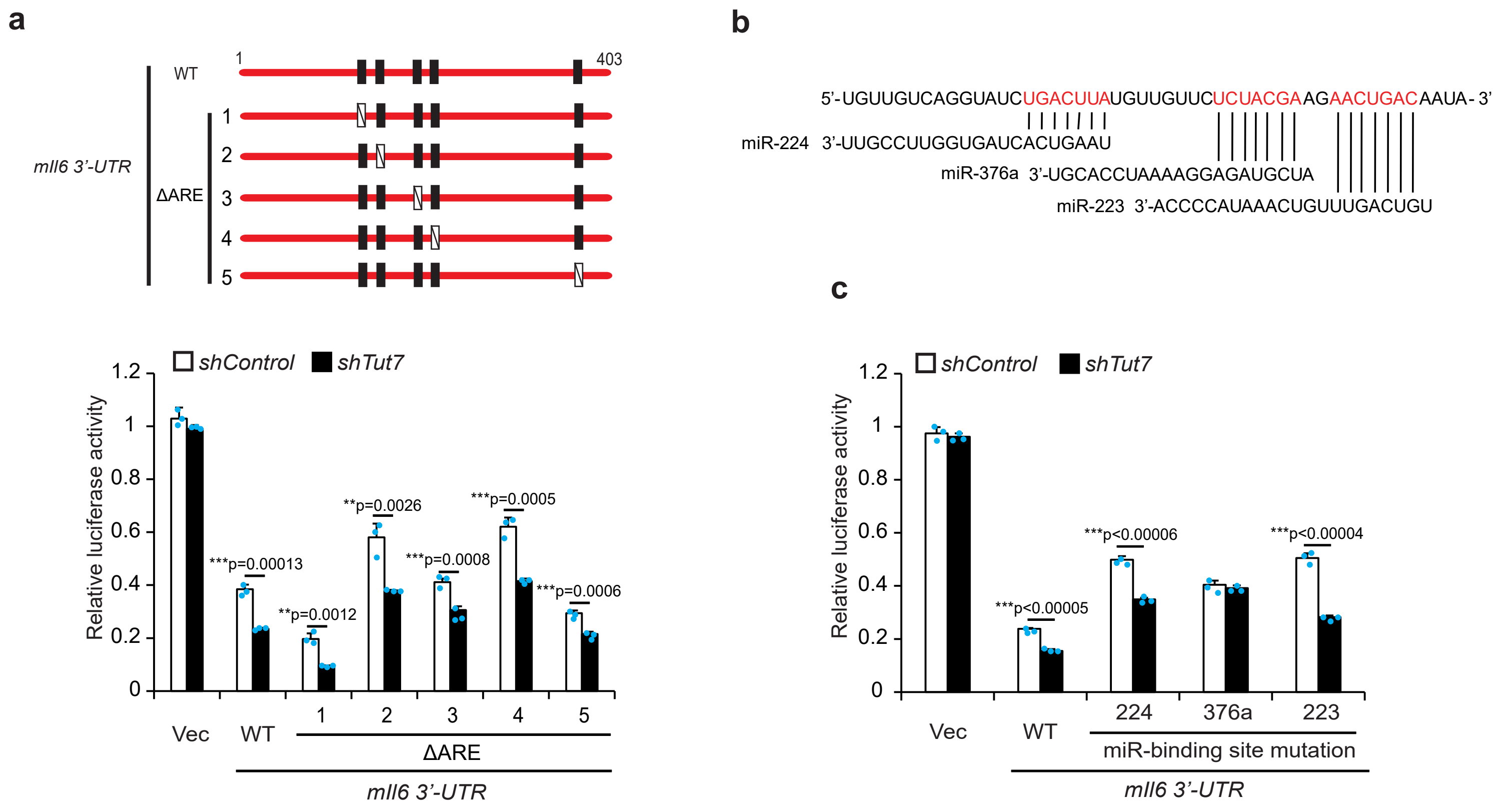

Supplementary Figure 5. TUT7-regulated *Il6* stability does not require ARE sites on its 3′-UTR.
(a) Top: Schematic diagram of the *firefly* luciferase reporter constructs containing full-length (WT) or AU-rich element mutation (AUUUAAGGGA) (ΔARE1-5 of *Il6* 3'-UTR). Bottom: Control and *shTut7*-expressing (*shTut7*) RAW 264.7 macrophages were co-transfected with *firefly* luciferase reporter constructs (WT and ΔARE1-5 of *Il6* 3'-UTR) and *TK-renilla* luciferase. At 48 hr after transfection, cells were treated with LPS (100 ng/ml) for 8 hr, followed by analysis of the luciferase activities as described previously. (b) Schematic diagram of predicted miRNAs and their putative binding sites on mouse *Il6* 3'-UTR. (c) Control and *shTut7*-expressing RAW 264.7 macrophages were co-transfected with *firefly* luciferase reporter plasmids containing wild-type or different *Il6* 3'-UTR mutants with deletion at individual miRNA-binding site as well as *TK-renilla* luciferase. Cells were treated with LPS (100 ng/ml) and luciferase activities were analyzed 8 hr later. Data presented are representative of three independent experiments with triplicates in each experiment (error bars, mean ± S.D.). The *p* values were obtained from two-tailed Student's *t*-test and are shown in the figure if $p < 0.05$. Source data are provided as a Source Data file.

Supplementary Figure 6, Related to Fig. 3

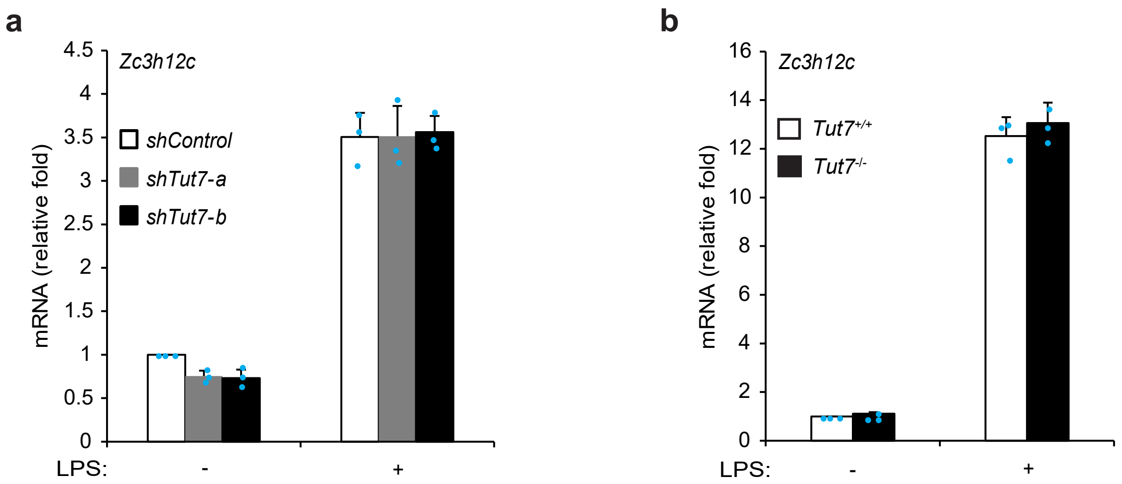

Supplementary Figure 6. TUT7 does not regulate *Zc3h12c* expression.
(a, b) Control and *shTut7*-expressing RAW 264.7 macrophages (a), and wild-type and *Tut7*$^{-/-}$ BMDMs (b) were stimulated with 100 ng/ml of LPS for 4 hr. The expression of *Zc3h12c* mRNA was analyzed by RT-qPCR. Data presented are representative of three independent experiments performed in triplicate (error bars, mean ± S.D.). Source data are provided as a Source Data file.

Supplementary Figure 7, Related to Fig. 4

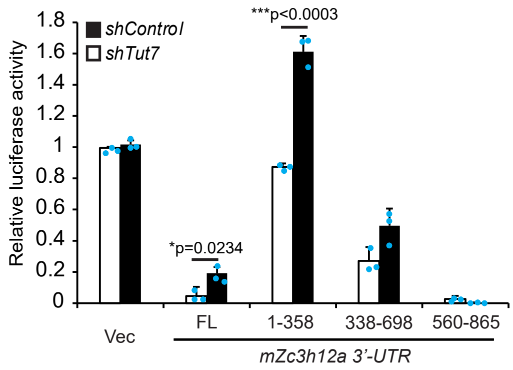

Supplementary Figure 7. TUT7 regulates *Zc3h12a* through its 3'-UTR$_{100\text{-}250}$.
Control and *shTut7*-expressing RAW 264.7 cells were co-transfected with *firefly* luciferase reporter constructs containing full-length (FL) or different truncated forms of *Zc3h12a* 3'-UTR as well as *TK-renilla* luciferase. Forty-eight hr later, transfected cells were stimulated with 100 ng/ml of LPS. Dual-luciferase activity assay was performed 8 hr after stimulation. Data presented are representative of three independent experiments with triplicates in each experiment (error bars, mean ± S.D.). The p values were obtained from two-tailed Student's t-test and are shown in the figure if $p < 0.05$. Source data are provided as a Source Data file.

Supplementary Figure 8, Related to Fig. 5

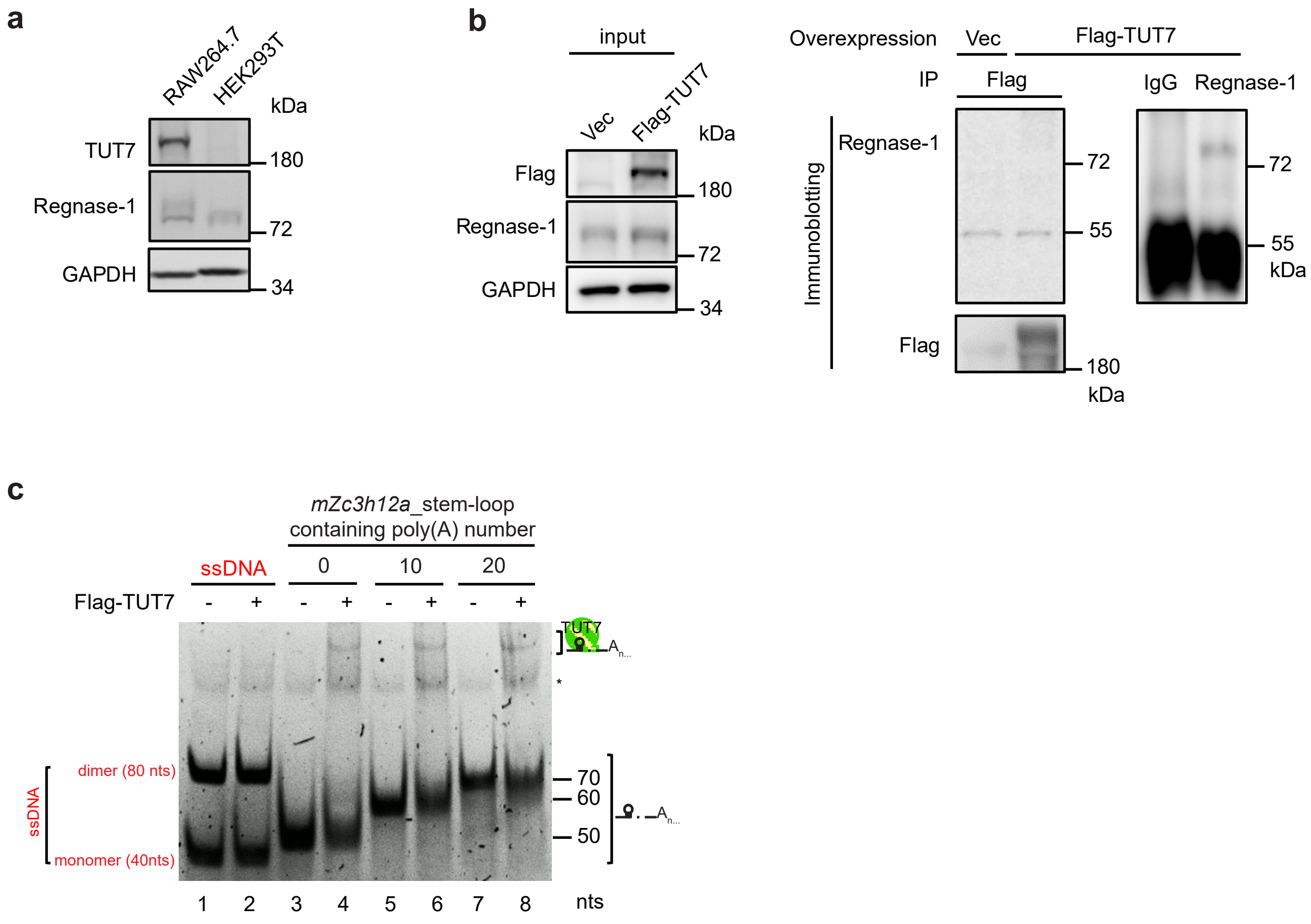

Supplementary Figure 8. TUT7 associates with *Zc3h12a* independent on the length of its poly(A) tails.
(a) RAW 264.7 and HEK293T cells were collected and lysed. TUT7 and Regnase-1 was analyzed by immunoblotting. (b) HEK293T cells were transfected with either vector or Flag-tagged TUT7 expression plasmid, and cell lysates were incubated with control IgG or protein A-agarose beads-conjugated anti-Flag or Regnase-1 antibody at 4°C for 4 hr. The immunocomplexes were analyzed by immunoblotting with the indicated antibodies. (c) The synthetic *Zc3h12a* RNAs containing stem-loop with various poly(A) tail lengths were incubated with immunopurified flag-tagged wild-type TUT7 at 37°C for 30 min. RNA samples were resolved on a 6% PAGE and visualized by SYBR Green II staining. Single-strand DNA (ssDNA) acts as a negatively binding control of TUT7. * represents the non-specific binding. Source data are provided as a Source Data file.

Supplementary Figure 9, Related to Fig. 6

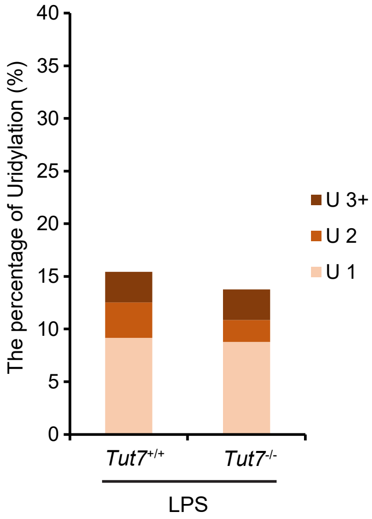

Supplementary Figure 9. *Il6* transcripts were analyzed by gene specific TAIL-Seq.
The frequency of uridylation on the 3' end of *Il6* was obtained from the experiments described in Fig. 6a. Uridylation frequency is the percentage of uridylated *Il6* reads out of the total number of *Il6* reads. Source data are provided as a Source Data file.

Supplementary Figure 10

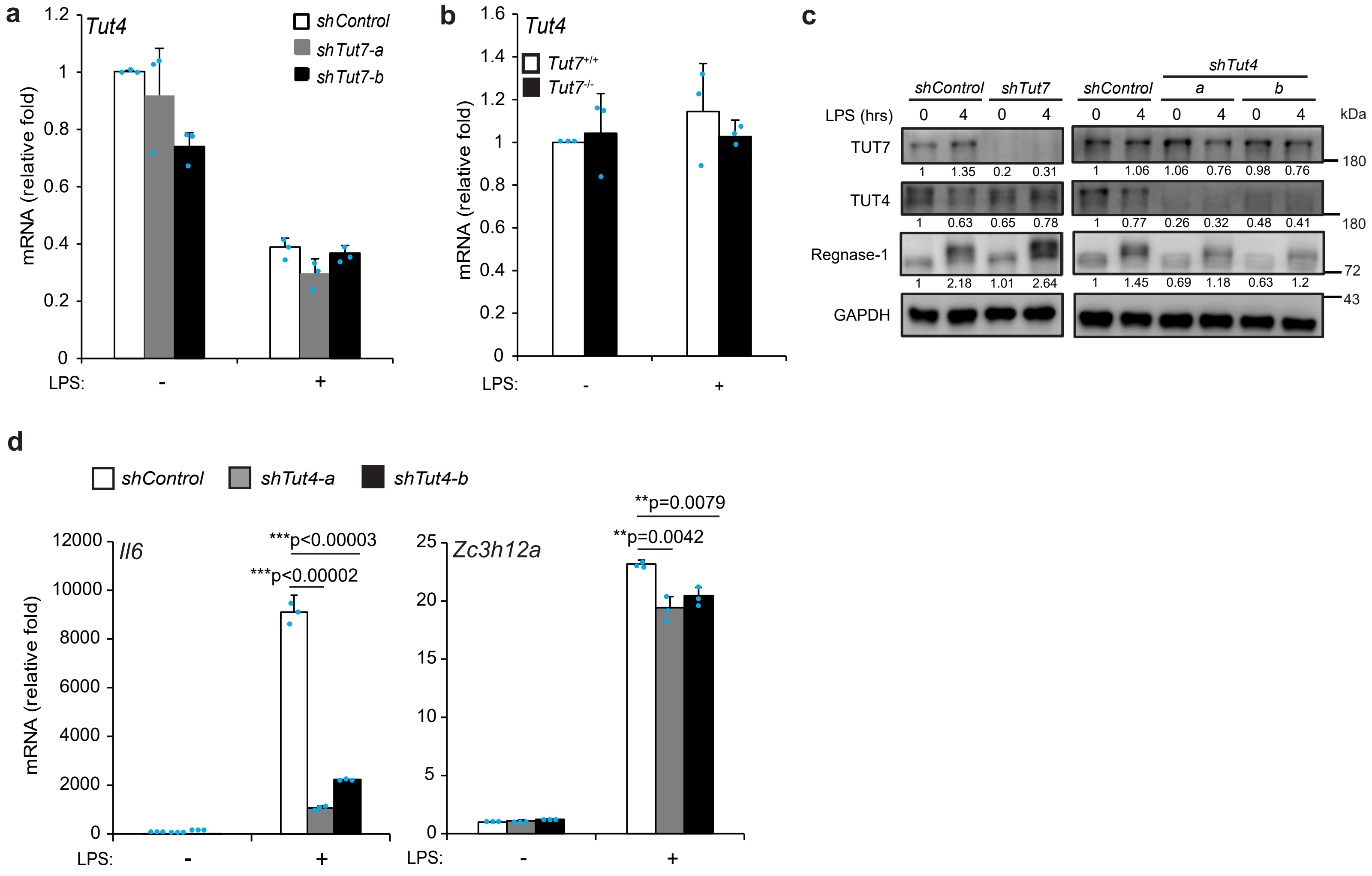

Supplementary Figure 10. Depletion of TUT4 decreases *Zc3h12a* expression in LPS-stimulated macrophages.
(a, b) Control and *shTut7*-expressing RAW 264.7 macrophages (a) and wild-type and *Tut7*$^{-/-}$ BMDMs (b) were treated with 100 ng/ml of LPS for 4 hr. The expression of *Tut4* mRNA was analyzed by RT-qPCR. (c) Control-, *shTut7*- and *shTut4*-expressing RAW 264.7 macrophages were challenged with 100 ng/ml of LPS for the indicated periods of time. The expressions of TUT7, TUT4, and Regnase-1 proteins were analyzed by immunoblotting. (d) Control and *shTut4*-expressing RAW 264.7 macrophages were stimulated with 100 ng/ml of LPS for 4 hr. The expression of *Il6* and *Zc3h12a* was analyzed by RT-qPCR. Data presented are representative of three independent experiments with triplicates in each experiment (error bars, mean ± S.D.). The *p* values were obtained from two-tailed Student's *t*-test and are shown in the figure if $p < 0.05$. Source data are provided as a Source Data file.

Supplementary Table 1

Complete blood counts of *Tut7*$^{+/+}$ and *Tut7*$^{-/-}$ mice

Parameter	*Tut7*$^{+/+}$	*Tut7*$^{-/-}$	P-value
RBC (M/uL)	10.54±0.54	10.22±0.58	0.41
HGB (g/dL)	15.88±0.57	15.35±0.79	0.28
WBC (K/uL)	10.33±2.60	6.89±2.31	0.07
Lymphocytes (%)	85.94±2.47	87.65±2.21	0.65
Granulocytes (%)	14.05±0.87	12.37±0.15	0.69

Data presented are the average values (mean ± SD) obtained from five *Tut7*$^{+/+}$ and *Tut7*$^{-/-}$ mice. Source data are provided as a Source Data file.

Supplementary Table 2. Downregulated innate immune-related genes in *Tut7*$^{-/-}$ mice compared to *Tut7*$^{+/+}$ mice upon LPS treatment.

ID	logFC	-Log10(PValue)	Description
Cd24a	-3.135596	5.421557049	CD24a antigen
Il23a	-2.526794	3.604987734	interleukin 23, alpha subunit p19
Il6	-1.984501	51.70215063	interleukin 6
Ccl17	-1.861305	3.662956589	chemokine (C-C motif) ligand 17
Igf2	-1.837911	1.717930791	insulin-like growth factor 2
Cd55	-1.72727	3.811074894	CD55 molecule
Il23r	-1.618184	1.794381129	interleukin 23 receptor
Cav1	-1.461788	30.28898137	caveolin 1
Havcr2	-1.329088	6.429341459	hepatitis A virus cellular receptor 2
Hmgb2	-1.304431	19.46858673	high mobility group box 2
Axl	-1.247568	33.02833109	AXL receptor tyrosine kinase

Cx3cl1	-1.2455	12.48041126	chemokine (C-X3-C motif) ligand 1
Il12b	-1.234514	12.91373427	interleukin 12b
Ciita	-1.204109	3.470063909	class II transactivator
Il12a	-1.16167	3.221540796	interleukin 12a
H2-Ab1	-1.109272	22.17037381	histocompatibility 2, class II antigen A, beta 1
Pvr	-1.098944	14.24350862	poliovirus receptor
Gbp8	-1.098314	5.060946887	guanylate-binding protein 8
Cdc42ep2	-1.077362	11.2014694	CDC42 effector protein (Rho GTPase binding) 2
Clec5a	-1.031005	9.373884692	C-type lectin domain family 5, member a
Clec4n	-1.02779	4.242482306	C-type lectin domain family 4, member n
H2-Aa	-1.02718	15.13981093	histocompatibility 2, class II antigen A, alpha
Cfh	-0.985688	4.053349108	complement component factor h
H2-Eb1	-0.984836	14.45052409	histocompatibility 2, class II antigen E beta
Clec4e	-0.937514	16.37023462	C-type lectin domain family 4, member e
Csf1	-0.88738	15.65079796	colony stimulating factor 1 (macrophage)
Matr3	-0.862964	6.246464825	matrin 3
Cd74	-0.814547	14.95222881	CD74 antigen (invariant polypeptide of major histocompatibility complex, class II antigen-associated)
Ipo7	-0.808549	5.19946776	importin 7

Mapkapk3	-0.801195	8.305054078	mitogen-activated protein kinase-activated protein kinase 3
Gfi1	-0.799736	3.393528076	growth factor independent 1
Ulbp1	-0.792979	2.269593044	UL16 binding protein 1
Il34	-0.786842	1.759112398	interleukin 34
Ifnb1	-0.773562	1.321670474	interferon beta 1, fibroblast
Gbp4	-0.759821	7.391136547	guanylate binding protein 4
Gbp7	-0.72904	6.524894573	guanylate binding protein 7
Syncrip	-0.722906	5.805839438	synaptotagmin binding, cytoplasmic RNA interacting protein
Stat5a	-0.710013	10.42422066	signal transducer and activator of transcription 5A
Il27	-0.709866	10.71025815	interleukin 27
Erbin	-0.68832	6.980924104	Erbb2 interacting protein
Ccl3	-0.684445	11.62584377	chemokine (C-C motif) ligand 3
Pspc1	-0.683911	2.900381304	paraspeckle protein 1
Tnf	-0.676368	10.39067318	tumor necrosis factor
Ptpn2	-0.667273	4.269843931	protein tyrosine phosphatase, non-receptor type 2
Ticam2	-0.653711	3.942509525	toll-like receptor adaptor molecule 2
Naip6	-0.63422	3.85727446	NLR family, apoptosis inhibitory protein 6
Kynu	-0.62817	1.678460844	kynureninase (L-kynurenine hydrolase)
Nlrp1b	-0.623351	1.663532706	NLR family, pyrin domain containing 1B
Tarm1	-0.619401	5.995655294	T cell-interacting, activating receptor on myeloid cells 1

Eif2ak2	-0.572986	3.987378793	eukaryotic translation initiation factor 2-alpha kinase 2
Pde12	-0.572702	3.328444107	phosphodiesterase 12
Parp14	-0.556341	4.007398583	poly (ADP-ribose) polymerase family, member 14
Kif5b	-0.556322	2.528442604	kinesin family member 5B
Ddx3x	-0.555085	3.763445519	DEAD/H (Asp-Glu-Ala-Asp/His) box polypeptide 3, X-linked
Ifih1	-0.553725	4.497651001	interferon induced with helicase C domain 1
Setd2	-0.551956	4.366841638	SET domain containing 2
Il18	-0.545008	4.555487938	interleukin 18
Otud4	-0.544814	2.647451562	OTU domain containing 4
Nlrp3	-0.540917	7.265137173	NLR family, pyrin domain containing 3
C3	-0.534549	7.174763754	complement component 3
Ptpn22	-0.522712	1.480604362	protein tyrosine phosphatase, non-receptor type 22 (lymphoid)
Vamp7	-0.521502	1.927807557	vesicle-associated membrane protein 7
Anxa1	-0.521458	5.808244421	annexin A1
Ptprs	-0.51944	2.659899831	protein tyrosine phosphatase, receptor type, S
Rps6kb1	-0.501165	1.915149066	ribosomal protein S6 kinase, polypeptide 1
Tnfaip3	-0.500157	6.358249128	tumor necrosis factor, alpha-induced protein 3

Oas2	-0.4913	5.639767256	2'-5' oligoadenylate synthetase 2
Trp53	-0.490689	4.359370541	transformation related protein 53
Pum2	-0.488137	2.866159039	pumilio RNA-binding family member 2
Ripk2	-0.479839	5.297307437	receptor (TNFRSF)-interacting serine-threonine kinase 2
Cnot7	-0.455605	2.371130522	CCR4-NOT transcription complex, subunit 7
Ddx60	-0.455225	2.14782721	DEAD (Asp-Glu-Ala-Asp) box polypeptide 60
Irak2	-0.423093	4.260395819	interleukin-1 receptor-associated kinase 2
Ptx3	-0.417714	1.430087123	pentraxin related gene
Cx3cr1	-0.413036	1.420295423	chemokine (C-X3-C motif) receptor 1
Pycard	-0.412247	3.056888143	PYD and CARD domain containing
Gbp5	-0.408944	3.362980352	guanylate binding protein 5
Itch	-0.405739	1.945764206	itchy, E3 ubiquitin protein ligase
Ccl4	-0.405721	4.459932336	chemokine (C-C motif) ligand 4
Nr1h3	-0.396951	1.571874339	nuclear receptor subfamily 1, group H, member 3
Trim30a	-0.396761	2.610211877	tripartite motif-containing 30A
Jak2	-0.395868	2.623423382	Janus kinase 2
Acod1	-0.387226	4.217437131	aconitate decarboxylase 1
Trim26	-0.382606	3.591936213	tripartite motif-containing 26
Herc6	-0.379875	2.063363487	hect domain and RLD 6
Tlr1	-0.37802	3.116442545	toll-like receptor 1

Stat5b	-0.369779	3.060244659	signal transducer and activator of transcription 5B
Rab20	-0.347454	1.586468946	RAB20, member RAS oncogene family
Map3k5	-0.340709	1.386843335	mitogen-activated protein kinase kinase kinase 5
Dhx9	-0.331155	1.382933383	DEAH (Asp-Glu-Ala-His) box polypeptide 9
Peli1	-0.32758	1.984949843	pellino 1
Cd36	-0.326664	1.576352146	CD36 molecule
Tlr3	-0.326588	1.63945389	toll-like receptor 3
Tlr2	-0.325378	3.015650802	toll-like receptor 2
Trim12a	-0.315185	1.993635232	tripartite motif-containing 12A
Syk	-0.314793	2.661299373	spleen tyrosine kinase
Hspd1	-0.290518	1.550802793	heat shock protein 1 (chaperonin)
Ube2k	-0.289548	1.56803693	ubiquitin-conjugating enzyme E2K
Aim2	-0.289385	1.643424219	absent in melanoma 2
Ccl5	-0.28319	2.258670106	chemokine (C-C motif) ligand 5
Cybb	-0.276479	1.452993681	cytochrome b-245, beta polypeptide
Gbp6	-0.276243	1.410450066	guanylate binding protein 6
Stx11	-0.272285	1.865810829	syntaxin 11
Sfpq	-0.271379	1.981463977	splicing factor proline/glutamine rich (polypyrimidine tract binding protein associated)
Irf1	-0.258622	1.9080788	interferon regulatory factor 1

Irgm1	-0.24082	1.664194608	immunity-related GTPase family M member 1
Sp110	-0.223047	1.556826228	Sp110 nuclear body protein
Tnip1	-0.21941	1.415031839	TNFAIP3 interacting protein 1
Cd14	-0.214713	1.367309852	CD14 antigen
Ccl9	-0.214454	1.51595478	chemokine (C-C motif) ligand 9
Cxcl16	-0.198174	1.320161239	chemokine (C-X-C motif) ligand 16

Supplementary Table 3. Upregulated innate immune-related genes in *Tut7*$^{-/-}$ mice compared to *Tut7*$^{+/+}$ mice upon LPS treatment.

**ID**	**logFC**	**-Log10(PValue)**	**Description**
Irf7	0.2097138	1.460260967	interferon regulatory factor 7
Hck	0.2246322	1.434773819	hemopoietic cell kinase
Dhx58	0.2289099	1.504684939	DEXH (Asp-Glu-X-His) box polypeptide 58
Capg	0.2428611	1.511222408	capping protein (actin filament), gelsolin-like
C1qb	0.2482706	1.800345117	complement component 1, q subcomponent, beta polypeptide
Actr3	0.2569312	2.00764736	ARP3 actin-related protein 3
Trem2	0.2575944	1.466372205	triggering receptor expressed on myeloid cells 2
Rpl13a	0.2633799	1.557301776	ribosomal protein L13A
Elf4	0.2671944	1.709339995	E74-like factor 4 (ets domain transcription factor)
Arf6	0.2999929	2.00478282	ADP-ribosylation factor 6

Tlr13	0.31681	1.407813884	toll-like receptor 13
Tnip3	0.3173188	2.634599721	TNFAIP3 interacting protein 3
Pik3ap1	0.3233569	2.902917121	phosphoinositide-3-kinase adaptor protein 1
Was	0.3266693	1.795528748	Wiskott-Aldrich syndrome
Ptpn6	0.3304218	2.674199993	protein tyrosine phosphatase, non-receptor type 6
C1qc	0.3379062	2.831293864	complement component 1, q subcomponent, C chain
Lgals3	0.3477593	3.119492954	lectin, galactose binding, soluble 3
Tnfaip8l2	0.3573362	1.55822057	tumor necrosis factor, alpha-induced protein 8-like 2
Trim28	0.3615663	2.350262131	tripartite motif-containing 28
Adam15	0.3758911	2.630999153	a disintegrin and metallopeptidase domain 15 (metargidin)
Stat2	0.3826317	3.744086942	signal transducer and activator of transcription 2
Trim56	0.4090441	2.112135731	tripartite motif-containing 56
Gpatch3	0.414884	1.416364384	G patch domain containing 3
Fcgr1	0.4212162	4.739669051	Fc receptor, IgG, high affinity I
Cd84	0.4257549	3.082176283	CD84 antigen
Cebpg	0.4361607	1.543761897	CCAAT/enhancer binding protein (C/EBP), gamma
Trim35	0.4432222	2.988112912	tripartite motif-containing 35

Lyn	0.460869	5.388152444	LYN proto-oncogene, Src family tyrosine kinase
Tmem173	0.4739144	2.616915911	transmembrane protein 173
Nos2	0.4803586	5.613983106	nitric oxide synthase 2, inducible
Mcoln2	0.4916815	4.337696988	mucolipin 2
Myo1f	0.4978802	4.198070509	myosin IF
Trim11	0.5022067	2.042147715	tripartite motif-containing 11
Ccl6	0.5273432	4.600546031	chemokine (C-C motif) ligand 6
Flot1	0.5285952	3.658334524	flotillin 1
Cfb	0.5463738	6.671568182	complement factor B
Klrk1	0.5672035	1.756835845	killer cell lectin-like receptor subfamily K, member 1
Serinc5	0.6085892	3.06862055	serine incorporator 5
Vav1	0.6333864	7.840941225	vav 1 oncogene
Vps26b	0.6391499	2.914760581	VPS26 retromer complex component B
Trim8	0.6391951	3.12960658	tripartite motif-containing 8
Nlrp1a	0.6719072	1.625785873	NLR family, pyrin domain containing 1A
Nrros	0.7004131	4.73267607	negative regulator of reactive oxygen species
Fbxo9	0.7252317	1.918022179	f-box protein 9
Ccl7	0.7470881	11.39347083	chemokine (C-C motif) ligand 7
Slamf1	0.7602543	3.311767027	signaling lymphocytic activation molecule family member 1

Pik3cd	0.7655641	11.30202983	phosphatidylinositol-4,5-bisphosphate 3-kinase catalytic subunit delta
Zyx	0.7752949	13.60505266	zyxin
Slamf8	0.8082164	6.124247053	SLAM family member 8
Fcna	0.8733257	2.593930952	ficolin A
Msrb1	0.8811139	8.832846406	methionine sulfoxide reductase B1
Evl	0.8902098	12.84713227	Ena-vasodilator stimulated phosphoprotein
Clec7a	0.9201076	10.32262726	C-type lectin domain family 7, member a
Rab11fip5	0.9420545	12.6469561	RAB11 family interacting protein 5 (class I)
Ly86	0.97787	16.89464429	lymphocyte antigen 86
Dusp10	0.9785667	1.573267822	dual specificity phosphatase 10
C1qa	0.99831	19.6063915	complement component 1, q subcomponent, alpha polypeptide
Tyro3	1.0846386	1.304189833	TYRO3 protein tyrosine kinase 3
Cd300lf	1.1083051	2.162536528	CD300 molecule like family member F
Rab7b	1.1551707	11.18568121	RAB7B, member RAS oncogene family
Lcn2	1.1578147	13.32710492	lipocalin 2
Esr1	1.3538468	1.391380823	estrogen receptor 1 (alpha)
Ccl8	1.4551744	6.416002475	chemokine (C-C motif) ligand 8
Rab43	1.5103638	30.91492272	RAB43, member RAS oncogene family
Ccr1	1.5378291	9.798548168	chemokine (C-C motif) receptor 1
S100a8	1.5670831	1.557707902	S100 calcium binding protein A8 (calgranulin A)
Padi4	1.8867563	1.78554161	peptidyl arginine deiminase, type IV

Arg2	2.1301855	4.80245293	arginase type II

Supplementary Table 4

Primer pairs for plasmid construction.

Tut7-NotI Forward	ATAAGAATGCGGCCGCTGGGAGATACAGCAAAACCTTAC
Tut7-SacI Reverse	GTTTAGAGCTCTCCTGGTGGGCTG
Tut7-SacI Forward	AGGAGAGCTCTGGCTGTGTCCAG
Tut7-ApaI Reverse	ATATGGGCCCTCATGTAAAAGCACTGCCTGGAG

h*TUT7* Forward	GGGAGATACAGCAAAACCTTACTTTGTGAAGC
h*TUT7*-ApaI Reverse	ATATGGGCCCTCATGTAAAAGCACTGCCTG
h*TUT7*_DADA Forward	CAAACAGAGTGCCCTTGCCGTCTGTATG
h*TUT7*_DADA Reverse	CATACAGACGGCAAGGGCACTCTGTTTG

IL-6 3'-UTR$_{56\text{-}104}$ Forward	CTAGTCTAGAAATATATCCTGTTGTCAGGTATCTG
IL-6 3'-UTR$_{56\text{-}104}$ Reverse	CTAGTCTAGACATATTGTCAGTTCTTCGTAGAG
IL-6 3'-UTR$_{104\text{-}224}$ Forward	CTAGTCTAGAGACAATATGAATGTTGGGACAC
IL-6 3'-UTR$_{104\text{-}224}$ Reverse	CTAGTCTAGACATTTCAAGTGACACTTCAT

miR-224 mutant Forward	GTCAGGTATCTGCCGTCTGTTGTTCTCTAC
miR-224 mutant Reverse	GTAGAGAACAACAGACGGCAGATACCTGAC
miR-376a mutant Forward	CTTATGTTGTTCTCGAAGCAGAACTGACAATATG
miR-376a mutant Reverse	CATATTGTCAGTTCTGCTTCGAGAACAACATAAG
miR-223 mutant Forward	GTTCTCTACGAAGAAATTAAAATATGAATGTTGGG
miR-223 mutant Reverse	CCCAACATTCATATTTTAATTTCTTCGTAGAGAAC

IL-6 3'-UTR$_{56-104}$_m1 Forward	CCGGAATTCATGAATTGCTAATTTAAATATGTTTTTAAAG
IL-6 3'-UTR$_{56-104}$_m1 Reverse	CCGGAATTCAACTTATACATTCCAAGAAACCATCTG
IL-6 3'-UTR$_{56-104}$_m2 Forward	CCGGAATTCAATGTTATATGTTATAGTTTTGAAATGATAA
IL-6 3'-UTR$_{56-104}$_m2 Reverse	CCGGAATTCCACTTCATAAAAATAATAAATATCAATCAT

*Zc3h12a*_EcoRI Forward	ccgGAATTCATGAGTGACCCTTGTGGAACG
*Zc3h12a*_XhoI Reverse	cggCTCGAGTTACTCACTGAGGTGCTGGGAC
*Zc3h12a*_ScaI Forward	aaaAGTACTGGTCTGAGCCGTACCCATTAC
*Zc3h12a*_3'-UTR_XhoI Reverse	cggCTCGAGGGCAATAGCTTTTTTTTTCTTTTAA
*Zc3h12a*_3'-UTR_XhoI Forward	cggCTCGAGGCCAGAAGGTGGCGCAA
*Zc3h12a*_3'-UTR_XbaI Reverse	gcTCTAGAGGCAATAGCTTTTTTTTTCTTTTAA
*Tnf*_CDS_EcoRI Forward	ccgGAATTCATGAGCACAGAAAGCA
*Tnf*_CDS_XhoI Reverse	cggCTCGAGTCACAGAGCAATGACTCC
*Tnf*_3’-UTR_XhoI Forward	ccgCTCGAGAAGGGAATGGGT
*Tnf*_3’-UTR_XhoI Reverse	ccgCTCGAGTCACATTTCTTTTCCAAGCGA

*Zc3h12a*_3’-UTR$_{1-358}$-XbaI Forward	gcTCTAGAGCCAGAAGGTGGCGCAAGGG
*Zc3h12a*_3’-UTR$_{1-358}$-XbaI Reverse	gcTCTAGACGTCCCAGGGACAGGGTG
*Zc3h12a*_3’-UTR$_{1-151}$-XbaI Reverse	gcTCTAGACGCGGGGCCAGAACCAAT
*Zc3h12a*_3’-UTR$_{100-250}$-XbaI Forward	gcTCTAGAGCAAGGGAATCCTCAAACCAAAG

*Zc3h12a*_3'-UTR$_{100-250}$-XbaI Reverse	gcTCTAGAGGATGGACGGCCTGACC
*Zc3h12a*_3'-UTR$_{210-358}$-XbaI Forward	gcTCTAGACTGTCATTTAAGGAGACGCTGC
*Zc3h12a*_3'-UTR$_{338-698}$-XbaI Forward	gcTCTAGAGGAGGCATGGGGGAGCAGAG
*Zc3h12a*_3'-UTR$_{338-698}$-XbaI Reverse	gcTCTAGACAGCCTGCCTGGCAATAGC
*Zc3h12a*_3'-UTR$_{560-865}$-XbaI Forward	gcTCTAGAGTATGCTGTGTACAGAGGC
*Zc3h12a*_3'-UTR$_{560-865}$-XbaI Reverse	gcTCTAGAGGCAATAGCTTTTTTTTTC

Supplementary Table 5

Primer pairs for RT-qPCR

Mouse *Cyclophilin* Forward	ATGTGCCAGGGTGGTGACTTT
Mouse *Cyclophilin* Reverse	TTGCCATCCAGCCATTCAGTC
Mouse *Il6* Forward	ACAAGAAAGACAAAGCCAGAGTC
Mouse *Il6* Reverse	ATTGGAAATTGGGGTAGGAAG
Mouse *Il12b* Forward	TGCAGATGAAGCCTTTGAAGA
Mouse *Il12b* Reverse	AACGCACCTTTCTGGTTACAC
Mouse *Il1b* Forward	GAACTCAACTGTGAAATGCCACC
Mouse *Il1b* Reverse	CCACAGCCACAATGAGTGATACT
Mouse *Tnf* Forward	GTCTACTGAACTTCGGGGTGATC
Mouse *Tnf* Reverse	TCCACTTGGTGGTTTGCTACG
Mouse *Nfkbia* Forward	TGCTGAGGCACTTCTGAAAGC
Mouse *Nfkbia* Reverse	CTGCGTCAAGACTGCTACACT
Mouse *Zc3h12a* Forward	TATCACAGACCAGCACATCCTTC
Mouse *Zc3h12a* Reverse	GTCTCGGTACGTGTCATTGGAG
Mouse *Zc3h12c* Forward	GTTGTAGTAGATGACGGTGAG
Mouse *Zc3h12c* Reverse	GTCAGGGCGGGACTGTTCTTT

Mouse *Tut4* Forward	AGAGTGAACTTCGGTCTCTTCC
Mouse *Tut4* Reverse	AACCATACAACCTCACTGAACATTC

Supplementary Table 6

Oligonucleotides used for cDNA library construction of TAIL-Seq.

Linker-1	CTGTAGGCACCATCAAT
p7_D701	CAAGCAGAAGACGGCATACGAGATCGAGTAAT GTGACTGGAGTTCAGACGTGTGCTCTTC
p7_D702	CAAGCAGAAGACGGCATACGAGATTCTCCGGA GTGACTGGAGTTCAGACGTGTGCTCTTC
p7_D703	CAAGCAGAAGACGGCATACGAGATAATGAGCG GTGACTGGAGTTCAGACGTGTGCTCTTC
p7_D704	CAAGCAGAAGACGGCATACGAGATGGAATCTC GTGACTGGAGTTCAGACGTGTGCTCTTC
p7_D705	CAAGCAGAAGACGGCATACGAGATTTCTGAAT GTGACTGGAGTTCAGACGTGTGCTCTTC
p7_D706	CAAGCAGAAGACGGCATACGAGATACGAATTC GTGACTGGAGTTCAGACGTGTGCTCTTC
p7_D708	CAAGCAGAAGACGGCATACGAGATGCGCATTA GTGACTGGAGTTCAGACGTGTGCTCTTC
p7_D709	CAAGCAGAAGACGGCATACGAGATCATAGCCG GTGACTGGAGTTCAGACGTGTGCTCTTC
p7_D710	CAAGCAGAAGACGGCATACGAGATTTCGCGGA GTGACTGGAGTTCAGACGTGTGCTCTTC
p7_D711	CAAGCAGAAGACGGCATACGAGATGCGCGAGA

	GTGACTGGAGTTCAGACGTGTGCTCTTC
p7_D712	CAAGCAGAAGACGGCATACGAGATCTATCGCT<br>GTGACTGGAGTTCAGACGTGTGCTCTTC
Zc3h12a_F1	GTGACTGGAGTTCAGACGTGTGCTCTTCCGATCT<br>tttaaatgaaaaaggttgacaaaataaa
Il6_F1	GTGACTGGAGTTCAGACGTGTGCTCTTCCGATCT<br>tgtttagactgtcttcaaacaaataaa
p5_D501	AATGATACGGCGACCACCGAGATCTACACTATAGCCT<br>ACACTCTTTCCCTACACGACGC
p5_D502	AATGATACGGCGACCACCGAGATCTACACATAGAGGC<br>ACACTCTTTCCCTACACGACGC
p5_D508	AATGATACGGCGACCACCGAGATCTACACGTACTGAC<br>ACACTCTTTCCCTACACGACGC
RT linker-1	ACACTCTTTCCCTACACGACGCTCTTCCGATCT<br>ATTGATGGTGCCTACAG

Supplementary Table 7

RNA oligomers used for RNA EMSA and competition assay.

Cy3-*Zc3h12a*-stem loop Wild-type	Cy3-ACAGGUCAGAAGUGAUCACCCUGUU<br>**GAUACACAUUGUAUC**UCUGUCAUUU
Zc3h12a-stem loop Wild-type	ACAGGUCAGAAGUGAUCACCCUGUU<br>**GAUACACAUUGUAUC**UCUGUCAUUU
Zc3h12a-stem loop St1m	ACAGGUCAGAAGUGAUCACCCUGUU<br>**GAAUGACAUUGUAUC**UCUGUCAUUU
Zc3h12a-stem loop St2m	ACAGGUCAGAAGUGAUCACCCUGUU<br>**GAUACACAUUCAUUC**UCUGUCAUUU
Zc3h12a-stem loop St(1+2)m	ACAGGUCAGAAGUGAUCACCCUGUU<br>**GAAUGACAUUCAUUC**UCUGUCAUUU
Il6-stem loop Wild-type	AAUAUAUCCUGUUGUCAGGUAUCUGACUUA<br>**UGUUGUUCUCUACGAAGAACUGACA**AUAUG

Fig. 1a

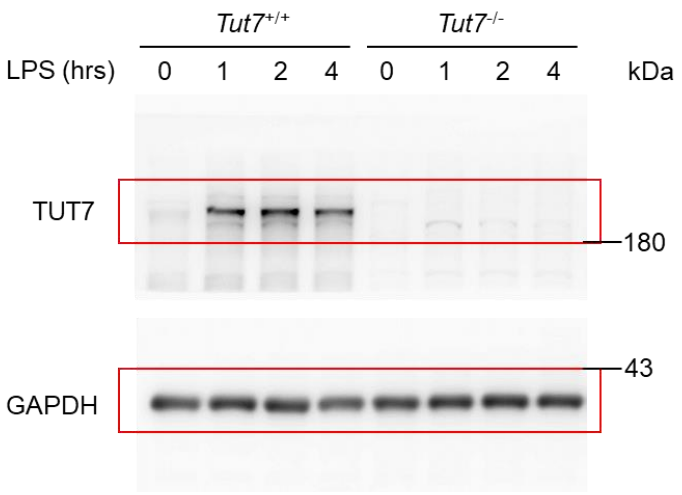

Fig. 1e

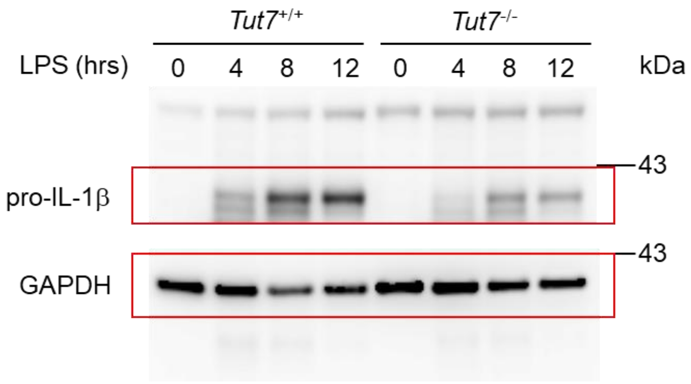

Fig. 1f

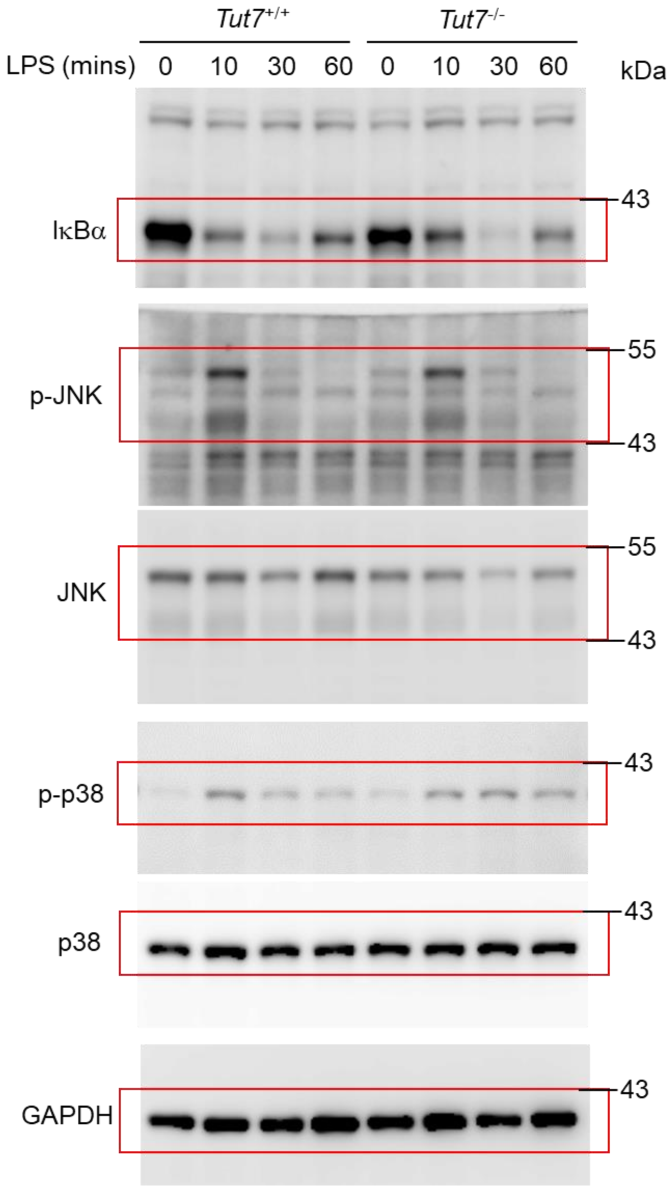

Fig. 2a

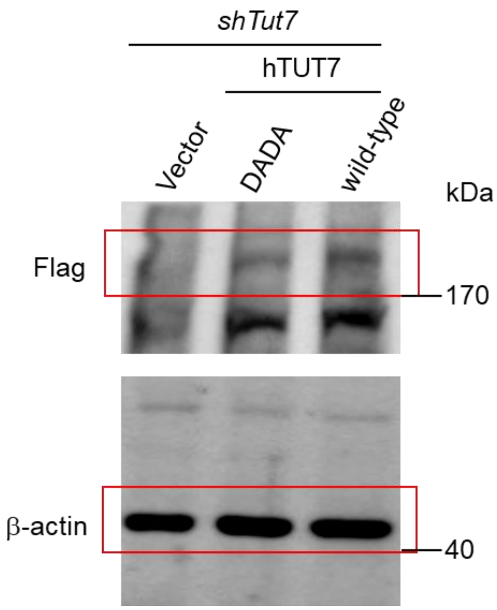

Fig. 3a

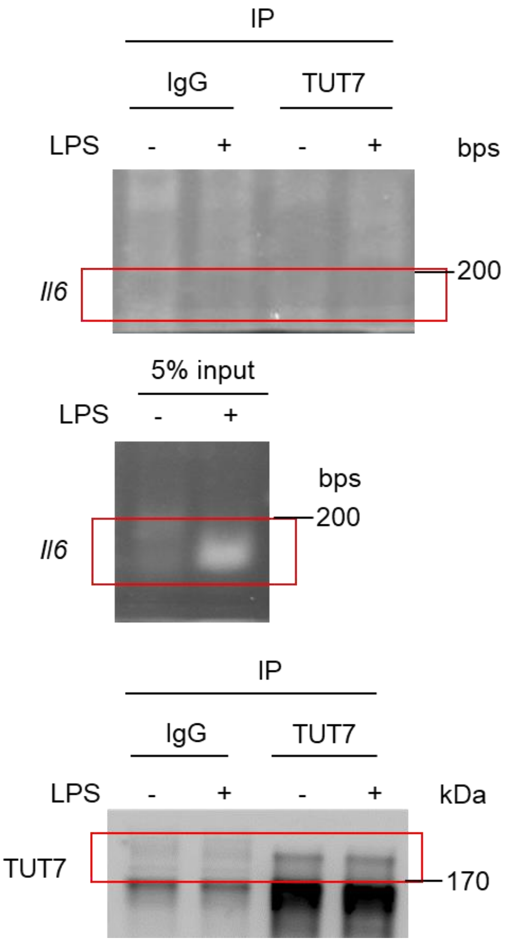

Fig. 3b

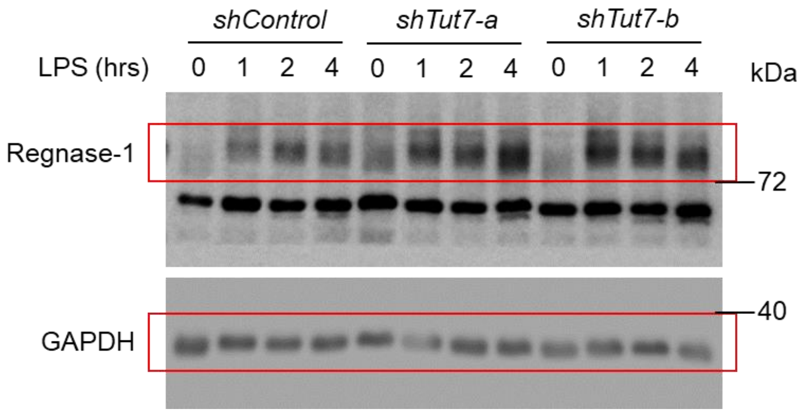

Fig. 3d

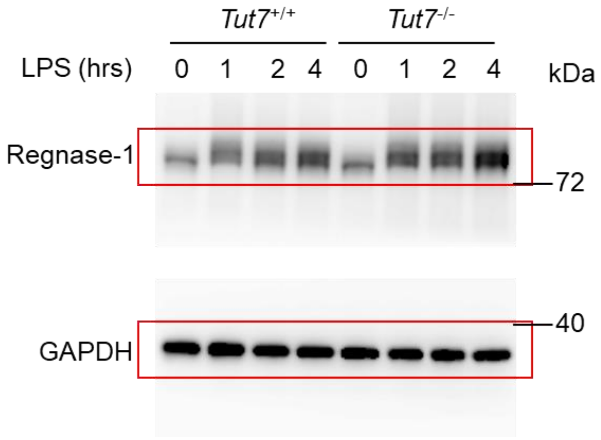

Fig. 3g

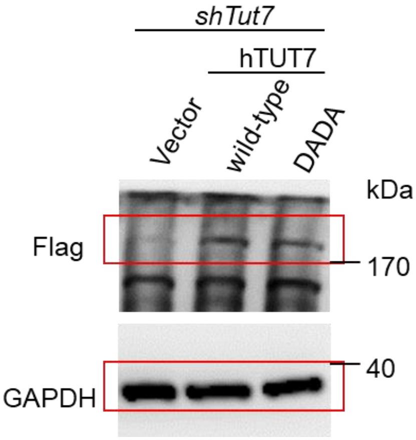

Fig. 4b

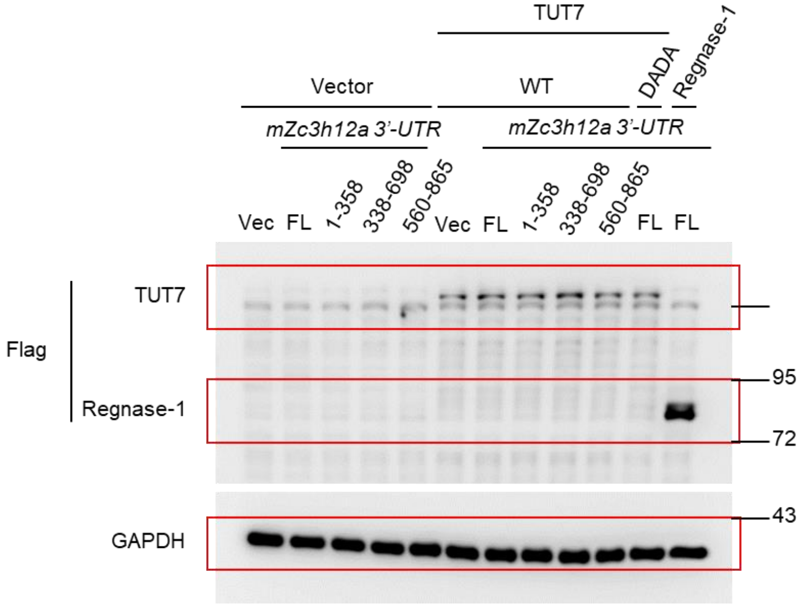

Fig. 4c

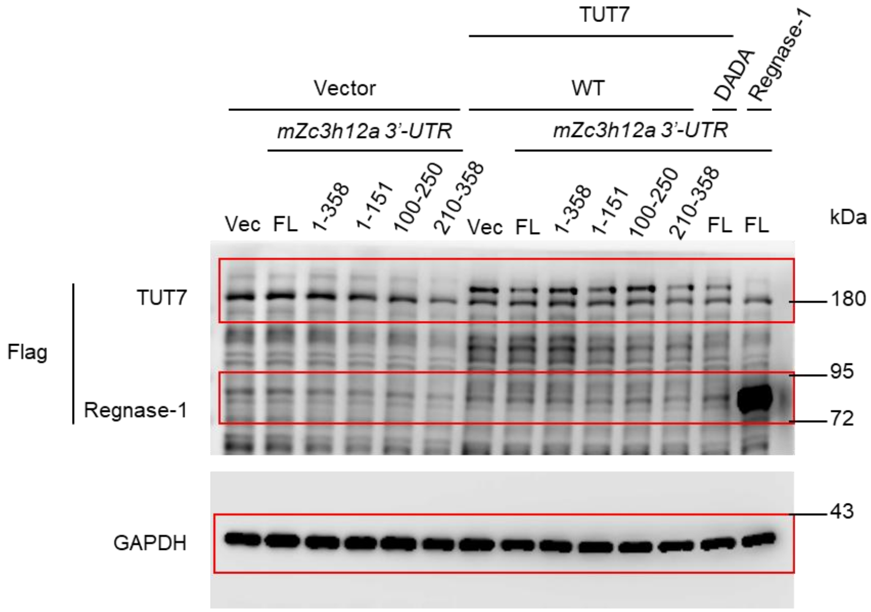

Fig. 4e

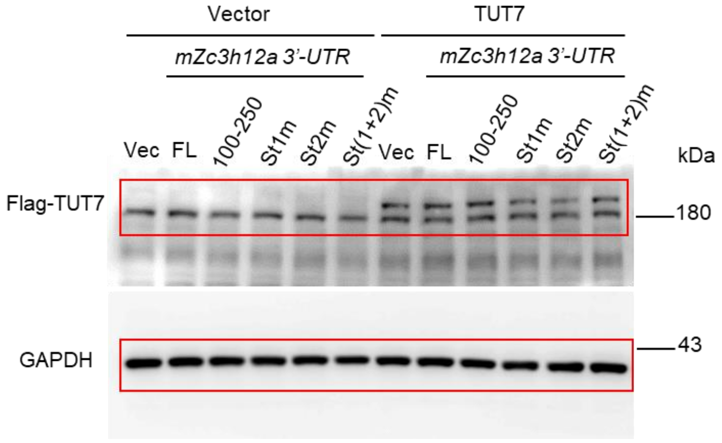

Fig. 5a

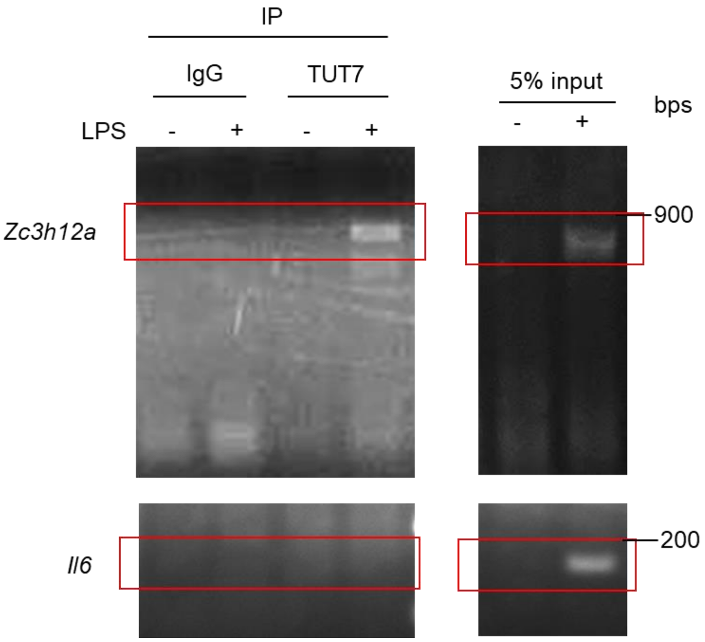

Fig. 5f (top panel)

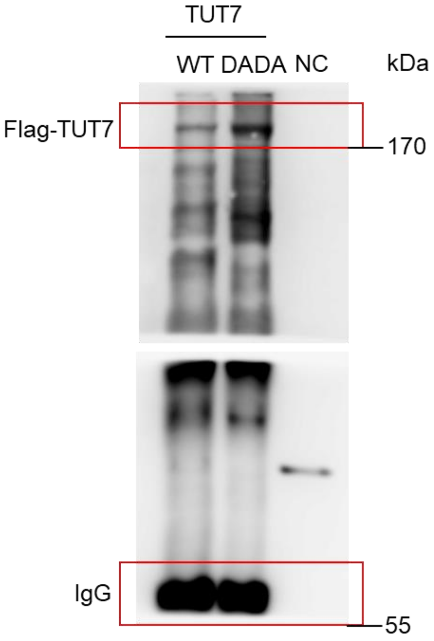

Supplementary figure 1a

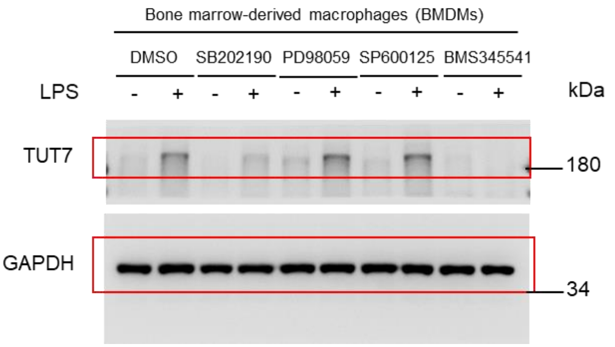

Supplementary figure 1b

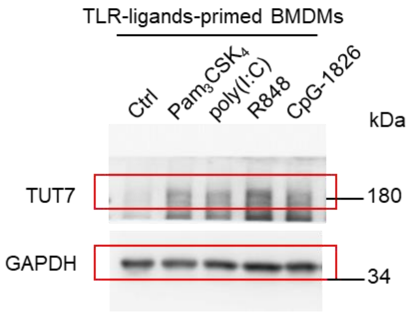

Supplementary figure 1c

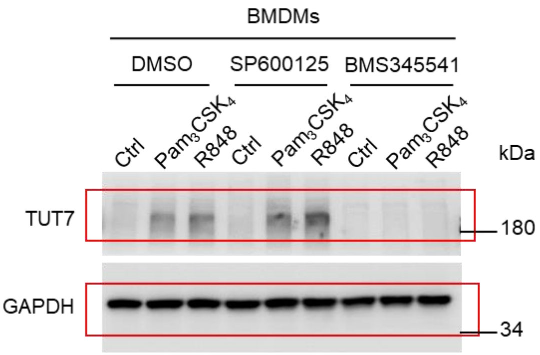

Supplementary figure 1d

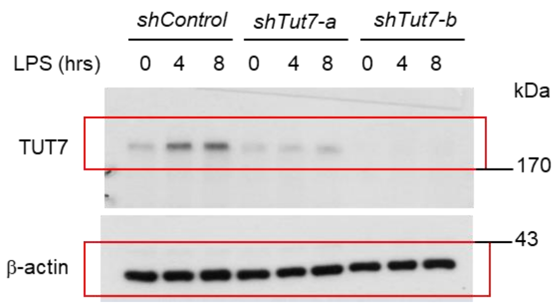

Supplementary figure 1g

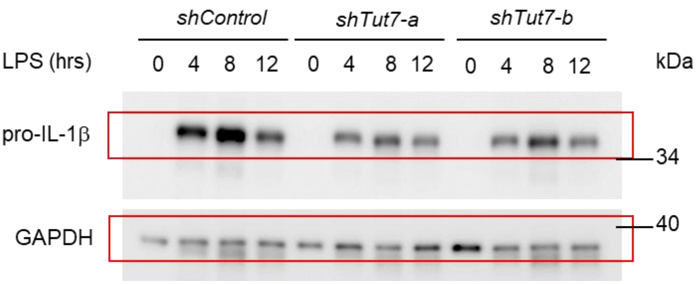

Supplementary figure 1i

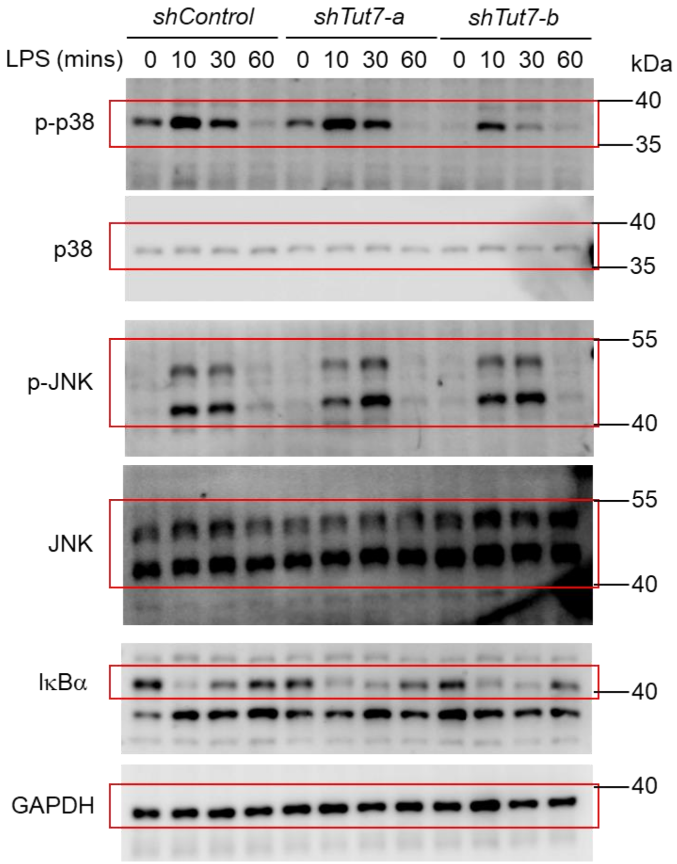

Supplementary figure 1j

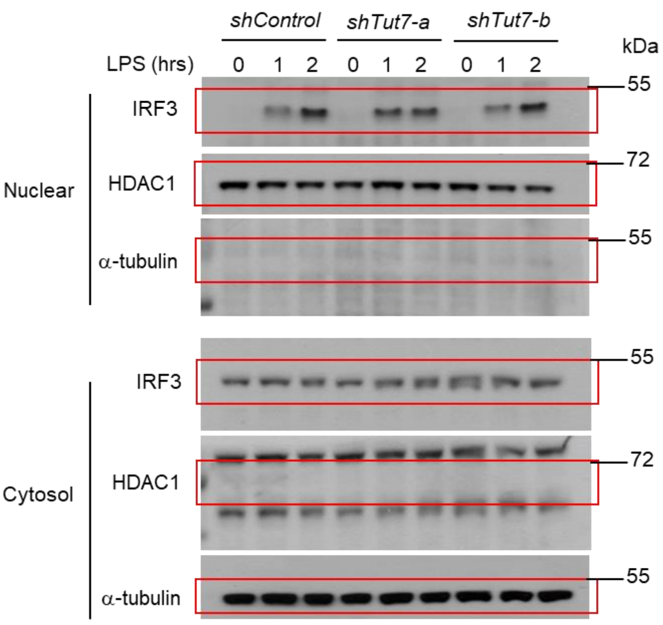

Supplementary figure 10c

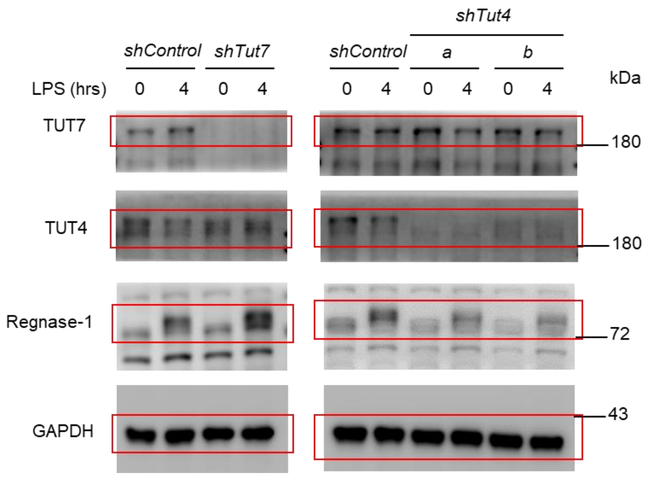

Supplement: Supplementary file 1 — Supplementary information [file 41467_2021_24177_MOESM1_ESM.pdf]
